# Spatiotemporal Modeling of Cholera, Uvira, Democratic Republic of the Congo, 2016−2020

**DOI:** 10.3201/eid3008.231137

**Published:** 2024-08

**Authors:** Ruwan Ratnayake, Jackie Knee, Oliver Cumming, Jaime Mufitini Saidi, Baron Bashige Rumedeka, Flavio Finger, Andrew S. Azman, W. John Edmunds, Francesco Checchi, Karin Gallandat

**Affiliations:** London School of Hygiene & Tropical Medicine, London, UK (R. Ratnayake, J. Knee, O. Cumming, W.J. Edmunds, F. Checchi, K. Gallandat);; Ministère de la Santé Publique, Division Provinciale de la Santé du Sud-Kivu, Zone de Santé d’Uvira, Uvira, Democratic Republic of the Congo (J.M. Saidi, B.B. Rumedeka);; Epicentre, Paris, France (F. Finger);; Johns Hopkins Bloomberg School of Public Health, Baltimore, Maryland, USA (A.S. Azman);; Geneva University Hospitals, Geneva, Switzerland (A.S. Azman)

**Keywords:** cholera, bacteria, spatiotemporal modeling, Democratic Republic of the Congo, disease clusters, outbreaks, prevention AND control, transmission dynamics, *Vibrio cholerae*

## Abstract

We evaluated the spatiotemporal clustering of rapid diagnostic test−positive cholera cases in Uvira, eastern Democratic Republic of the Congo. We detected spatiotemporal clusters that consistently overlapped with major rivers, and we outlined the extent of zones of increased risk that are compatible with the radii currently used for targeted interventions.

Cholera outbreaks affect communities that lack access to safe water and adequate sanitation (*1*). Spatiotemporal clustering patterns of cholera indicate a high risk of transmission to the neighboring households of new cases (*2*,*3*). Case-area targeted interventions (CATI), consisting of early, multisectoral response within a 100–500-meter radius around case-households, have been proposed to attenuate clustered transmission (*4*). CATIs, driven by water, sanitation, and hygiene interventions, played a major role in response strategies in Haiti and Yemen, and CATIs including oral cholera vaccination helped suppress outbreaks after vaccination campaigns in Cameroon (*5*,*6*). In the Democratic Republic of the Congo (DRC), health officials evaluated water, sanitation, and hygiene targeting strategies within 500 meters around households with cholera cases (*7*). In Kalemie, DRC, and N’Djamena, Chad, researchers estimated a 200-meter zone of increased risk of infection around cholera cases in the first 5 days (*2*). As CATIs become part of routine practice (*4*,*5*), more insight is needed in delineating the spatiotemporal risk zones required to achieve a substantive effect on transmission.

In Uvira, a city in eastern DRC affected by protracted conflict, population displacement, and flooding, cholera is endemic, and stable transmission is punctuated by seasonal outbreaks (*8*). Citywide interventions include an ongoing piped water supply program with household tap installation beginning in late 2019 (*9*) and mass vaccination in mid-2020 (*10*). Using an enhanced surveillance system with rapid diagnostic testing (RDT), we investigated the location, timing, and prediction of clusters to identify outbreaks earlier and trigger early response. We estimated the extent of spatiotemporal zones of increased risk around cases as a proxy for the ideal radius of CATIs.

## The Study

We analyzed suspected cases of cholera during 2016–2020 in patients at cholera treatment centers managed by the Uvira Health Zone. Beginning in April 2016, rectal swab samples were collected from suspected cases and RDT tested (Crystal VC O1/O139; Arkray Inc., https://www.arkray.co.in) after a 6-hour enrichment in alkaline peptone water. We classified cases by avenue of residence (i.e., enumeration areas of mean size 1,177 [range 180–5,711] based on 2017 population sizes) (town of Uvira census data, 2018, unpub. data). We used 2 methods to evaluate spatiotemporal clustering. The space–time scan statistic describes local clustering, where cases exceed expected density within a given area, to identify spatiotemporal clusters and assign relative risk comparing observed versus expected cases inside and outside the cluster (*11*). To assess capacity for early detection of outbreaks, we simulated real-time detection by scanning prospectively (using few cases) and compared the delay with retrospective scanning (using more cases). We calculated the proportion of years that avenues were included in clusters during 2016–2020. The tau statistic () describes global clustering, or the overall tendency for cases to occur near other cases in time and space (*12*), by using a relative risk of an individual in the population within a given distance band (i.e., 100–150 meters) from an incident case, compared with the risk for any individual in the population, becoming a potentially transmission-related case. This statistic suggests the geographic and temporal extents of increased infection risk. We defined the high-risk and elevated-risk zones as the radius where the moving average’s lower 95% CI (high risk) and point estimate (elevated risk) cross 1.0 for ≥30 consecutive meters. We based the main analyses on enriched RDT-positive cases. We conducted sensitivity analyses using suspected cases, and given the use of enumeration areas, using simulated household locations (Appendix). We carried out analyses in R software v.4.1.2 (The R Foundation for Statistical Computing, https://www.r-project.org) by using the rsatscan v.1.0.5 (combined with SaTScan v.10.0.2) and IDSpatialStats v.0.3.12 packages. 

**Figure 1:**
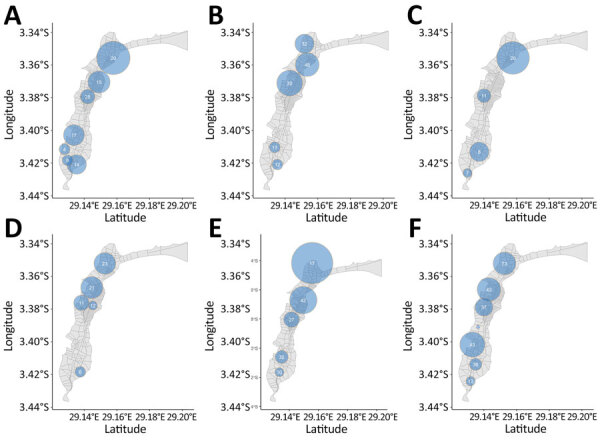
Spatial distribution of spatiotemporal clusters of rapid diagnostic test–positive cholera cases at the avenue level, Uvira, Democratic Republic of the Congo, 2016−2020. A: 2016, B: 2017, C: 2018, D: 2019, E: 2020, F: 2016—2020. Clusters have a relative risk >1 (p<0.05). The sizes of the light blue circles depict the spatial radius and the numbers of cases are shown inside the circles.

**Figure 2:**
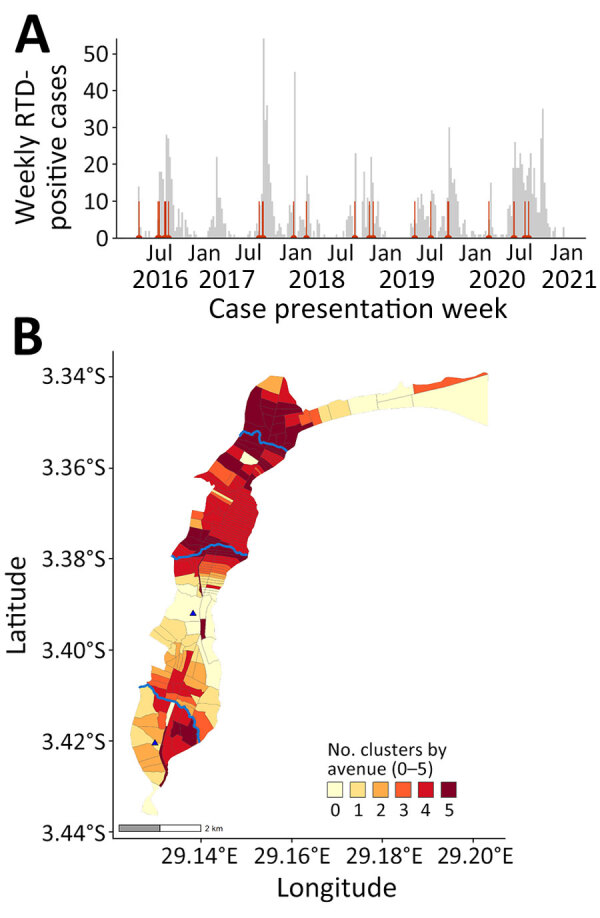
Epidemic curve and cluster persistence in study of spatiotemporal modeling of cholera, Uvira, Democratic Republic of the Congo, 2016−2020. A) Epidemic curve shows weekly numbers of RDT-positive cholera cases based on week of onset and start dates of 26 clusters (red vertical lines). B) Cluster persistence within avenues for RDT-positive cases showing the number of years affected by clustering within avenues and proximity to rivers (blue lines, top to bottom: Kalimabenge River, Mulongwe River, Kanvinvira River). Blue triangles indicate cholera treatment center (top) and unit (bottom). RDT, rapid diagnostic test.

Among 5,447 suspected cases, 3,456 (63.4%) were tested and 1,493 (43.2%) were RDT positive. We detected 26 significant spatiotemporal clusters (Table). Mean cluster radius was 652 (range 308–1582) meters, mean size was 20 (range 4–48) cases, and mean duration was 24.8 (range 1–58) days. Clustering occurred in similar locations annually (Figure 1). The first day of a retrospectively detected cluster usually anticipated a seasonal outbreak within 1 week, except for 2016 and 2017, when few cases were RDT tested (Figure 2, panel A). The median delay to the early outbreak signal was 1 day (interquartile range 0–3, maximum 23 days), and median size at signal detection was 3 cases (interquartile range 2–7, maximum 21 cases). Large clusters persisted across 2016–2020 and overlapped with major rivers in north-central and southern Uvira (Figure 2, panel B). We observed no changes in cluster locations in 2019, after household tap implementation began (Figures 1, panels D, E). Sensitivity analysis of suspected cases found more clusters (n = 32) in similar locations with similar mean radii (668 [range 331–1,557] meters), larger mean size (42 [range 6–130] cases), and longer duration (27.8 [range 1–59] days) (Appendix Table 2, Figure 5).

**Table:** Statistically significant spatiotemporal clusters of RDT-positive cholera cases detected through annual scanning at the avenue level, Uvira, Democratic Republic of the Congo, 2016−2020*

Year	No.	Cases observed: expected	Population at risk	RR†	Cluster radius, meters	Cluster start date	Cluster duration, d	Signal delay, d‡	Size at signal, no. cases
2016	1	20:1	30,553	20.9§	1,140	Aug 5	18	8	11
	2	28:3	34,232	10.5§	497	Jun 25	48	0	2
	3	17:1	30,758	13.8§	717	Jul 22	23	5	12
	4	15:1	31,240	11.9§	758	Jun 29	23	1	4
	5	4:0	6,579	344.4§	376	Apr 9	1	0	3
	6	14:2	30,082	8.8§	668	Jul 21	30	0	3
	7	9:1	27,452	12.6¶	368	Jul 26	14	3	4
2017	1	48:4	51,012	13.0§	811	Aug 7	40	2	2
	2	32:2	43,992	16.4§	657	Aug 20	23	1	13
	3	32:4	49,794	7.7§	880	Aug 23	44	0	2
	4	13:1	51,016	16.4§	378	Dec 24	7	0	2
	5	12:2	50,635	7.6¶	368	Aug 23	15	12	2
2018	1	20:1	28,884	26.6§	1,116	Oct 26	13	6	9
	2	11:1	31,204	22.7§	475	Feb 13	7	0	3
	3	8:0	25,148	40.6§	662	Aug 28	3	0	4
	4	7:0	17,345	18.6¶	308	Nov 10	10	1	3
2019	1	23:1	33,751	18.6§	743	Sep 10	18	1	7
	2	21:3	33,162	9.0§	755	Sep 7	35	0	12
	3	12:1	16,210	12.3§	309	Apr 27	29	1	2
	4	11:1	16,495	13.2§	527	Sep 7	24	0	2
	5	6:0	15,001	27.8¶	368	Jun 30	6	0	2
2020	1	42:6	60,378	7.8§	1,048	Jul 29	58	2	3
	2	27:3	42,423	8.7§	599	Jul 15	46	23	21
	3	17:1	56,029	19.1§	1,582	Feb 20	9	0	2
	4	30:5	63,207	6.5§	343	May 30	46	2	6
	5	32:6	63,593	5.8§	501	Jun 1	55	4	6

*RDT, rapid diagnostic test; RR, relative risk. †p values indicate the statistical significance of clusters derived from Monte Carlo simulations. ‡Signal delay indicates the number of days between retrospective detection date with all available data and the earliest prospective detection date. §p<0.001. ¶p<0.05.

**Figure 3:**
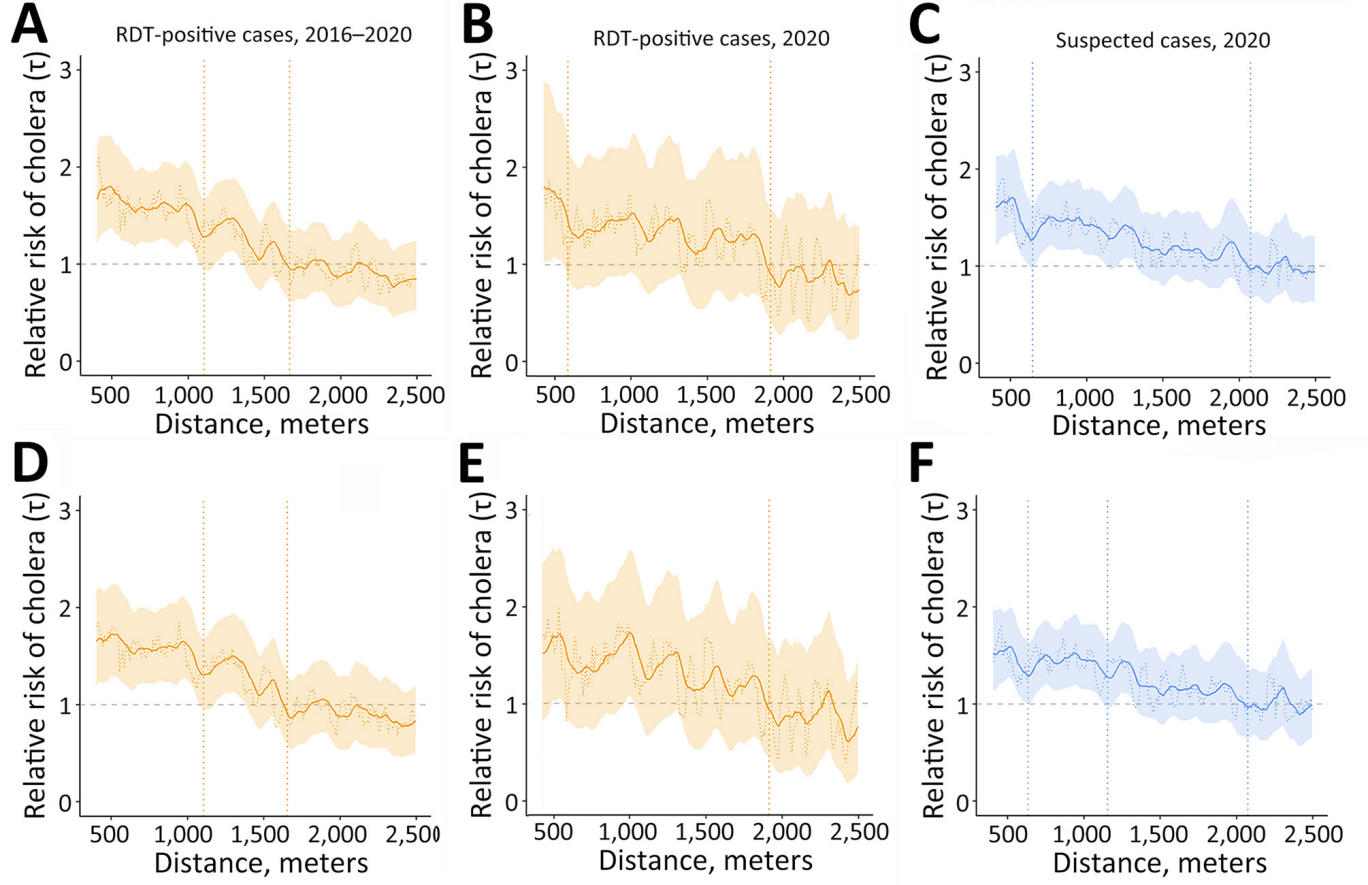
Moving average estimates for RDT-positive and suspected cholera cases in study of spatiotemporal modeling of cholera, Uvira, Democratic Republic of the Congo, 2016−2020. Moving average estimates of (relative risk) and 95% CIs (shading) are shown with point estimates (dashed horizontal lines) for days 0–4 (panels A–C) and days 1–4 (panels D–F), for RDT-positive cases (orange) and suspected cases (blue), using 1,000 bootstrap samples. The vertical dashed lines indicate the spatial extent of the zone of high-risk where the lower 95% CI crossed 1.0 for ≥30 meters consecutively (first line) and zone of elevated risk where the point estimate crossed 1.0 for ≥30 meters consecutively (second line). RDT, rapid diagnostic test.

In 2016–2020, within 5 days after cases began, the high-risk zone extended to 1,105 meters, and risk remained elevated up to 1,665 meters (maximum moving average τ = 1.8, 95% CI 1.4–2.3) (Figure 3, panel A). During days 1–4, which is more realistic for response, risk zones remained similar (Figure 3, panel D). In 2020, the high-risk zone extended to 585 meters and risk remained elevated up to 1,915 meters (τ = 1.8, 95% CI 1.0–2.9) (Figure 3, panel B). During days 1–4, the risk zones were 425 meters (high risk) and 1,915 meters (τ = 1.7, 95% CI 1.1–2.6) (Figure 3, panel E). Results were similar when we used simulated household locations (during days 0–4) with a moving average τ≥2.0 at 75–275 meters (τ = 2.4, 95% CI 1.7–3.3) and high-risk zone radius (1,415 meters) (Appendix Table 1, Figure 4). Annual results showed lower high-risk (425 meters, except 2017, when it was 875 meters) and elevated (1,125–1,485 meters) zone ranges and no discernable changes after 2019, when household tap implementation began (Appendix Figure 6). Using suspected cases from 2020, the trends remained similar (Figure 3, panels E, F).

## Conclusions

We detected spatiotemporal clustering of cholera outbreaks during 2016–2020 in Uvira, DRC, that could inform early mitigation of seasonal outbreaks. The clustering methods produced aligned results compatible with a high-risk radius of ≤500 meters, as previously used for CATI in DRC (*7*,*13*) and similar to clustering in Matlab, Bangladesh, and coastal Sabah, Malaysia (500 meters, ≈5 days after cases began) (*3*,*14*). For RDT-positive cases within 5 days after cases began, we estimated a 1,105-meter high-risk radius, showing that a ≤1,000-meter risk window is optimal. Scan statistics detected a similar mean cluster radius of 650 meters. The simulated real-time scanning usually signaled an outbreak with a 1-day median delay, which would enable early control.

We used enriched RDT-positive cases to increase specificity, but among study limitations is that we relied on medically attended cases at a cholera treatment center, biasing toward severely dehydrated case-patients and against milder cases. The spatial resolution misses case-pair distances <420 meters, where 5% of distances fell, although simulation of household locations showed similar trends with even higher across smaller radii. Circular scan statistics have reduced sensitivity to outline the shape of elliptical clusters (potentially along Uvira’s coastline), but detection appeared unaffected (*11*).

Conspicuously, the clusters endured annually and overlapped with Uvira’s 3 major rivers. According to surveys in 2016, 2017, and 2021, households in those clusters commonly use rivers as a primary water source (K. Gallandat et al., unpub. data) because piped water has remained inconsistent (*15*). Combined with the high population density and inadequate sanitation, close-contact, fecal–oral transmission is amplified, producing recurrent clustering. Preventive measures, including piped water and vaccination, could be reinforced in cluster locations. CATI could address containment for new cases in less affected areas to prevent larger outbreaks. Because lakeside cities like Uvira may regularly seed regional outbreaks, targeted disease control strategies may bring substantial public health benefits.

Appendix More information for spatiotemporal modeling of cholera, Uvira, Democratic Republic of the Congo, 2016−2020.
