# Supplementary material for: Spatiotemporal Modeling of Cholera, Uvira, Democratic Republic of the Congo, 2016−2020
Source: Emerg Infect Dis. 2024 Aug;30(8):1677–82. doi: 10.3201/eid3008.231137 (PMC11286051; doi:10.3201/eid3008.231137)
Supplement: Appendix — More information for spatiotemporal modeling of cholera, Uvira, Democratic Republic of the Congo, 2016−2020. [file 23-1137-Techapp-s1.pdf]

Article DOI: http://doi.org/10.3201/eid3008.231137

EID cannot ensure accessibility for supplementary materials supplied by authors. Readers who have difficulty accessing supplementary content should contact the authors for assistance.

Spatiotemporal Modeling of Cholera, Uvira, Democratic Republic of the Congo, 2016–2020

Appendix

Methods for Local and Global Clustering Statistics

Local Clustering to Identify Recurrent Locations and Timing of Seasonal Outbreaks

We used the space-time scan statistic to retrospectively detect the presence and location of spatiotemporal clusters. We conducted the analysis for the entire period (2016–2020) and according to each year. A relative risk (RR) compares the observed versus expected number of cases inside and outside of a cluster. Poisson distribution of the cases per avenue (or street) was assumed. To find the most likely cluster, candidate clusters were ordered according to a log-likelihood ratio (LLR), where the cluster with the largest LLR is the least likely to be caused by chance and, therefore, is the most likely cluster. The significance of each cluster was evaluated by using Monte Carlo simulation to compare the original dataset with 999 random replicates produced under the null hypothesis.

We examined the entire dataset (i.e., a retrospective scan). We restricted the temporal and spatial windows to capture brief periods (7–60 days) and a radius that included ≤10% of the population at risk. To capture clustering that persisted across years, we also used a longer temporal window (7–365 days) for 2016–2020.

To explore whether the space-time scan statistic produced signals that preceded outbreaks, we conducted prospective scans of each of the clusters that were detected retrospectively. This was done to detect the earliest warning sign that indicated when that cluster would have first been detected. We simulated repeated prospective scans on the retrospective cluster start day and each successive day (up to 4 weeks later). We calculated the median and

interquartile range for the delay between when the prospective scan would have first detected the cluster and the date produced by the retrospective scan, which used more case data. We also calculated the median and interquartile range for the cluster size at first detection. We visualized the timing of the first day of each retrospective cluster on an epidemic curve. To explore when cholera transmission predominated, we calculated the proportion of years that the avenue was included in any cluster during 2016–2020, ranging from 0 (not included in any cluster) to 5 (included in a cluster every year) (*1*).

Methods for Space-Time Scan Statistic

For a given cylinder consisting of a radius centered on an avenue centroid and height of the temporal window of interest, *c* is the observed number of cases inside the cylinder, *E[c]* is the expected number of cases for any given cylinder, and *C* is the total number of cases in Uvira (*2*). RR is calculated as:

$$RR = \frac{\frac{c}{E[c]}}{\frac{(C - c)}{(C - E[c])}}$$

During the scan, a circular scanning window with varying radii and duration moves over the geographic area so that each avenue centroid is at the center of several candidate clusters with different radii and heights. At each cylinder location, the number of cases inside the cylinder is compared with the expected number under a null hypothesis of no clustering (i.e., cases are randomly distributed). To find the most likely cluster, candidate clusters are ordered by the LLR and evaluated by using Monte Carlo simulation as previously described.

Global Clustering to Inform Risk Boundaries

We estimated the tau (τ) statistic for the entire period (2016—2020) and annually to quantify the spatial extent of the risk zone around an index case (*3*). Because the dataset only contained the date of the visit to the cholera treatment center/cholera treatment unit as opposed to the date of symptom onset, this statistic represented the risk of developing medically attended disease, which we assumed indicated severe dehydration and diarrhea compared with mild dehydration and diarrhea. This approach defines clustering according to how likely any pair of cases are potentially transmission-related within a given distance between the cases. Accordingly, we first classified each pair of cases as potentially transmission-related if their

dates of case presentation were within 0–4 days of each other (≈1 serial interval) (*4*). τ is the RR that a person in the population within a given distance (d_1, d_2) band (e.g., 100 m, 150 m) from an incident case becomes a potentially transmission-related case compared with the risk for any person in the population becoming a potentially transmission-related case. A τ value >1 indicates evidence of clustering within the given distance band.

As we lacked individual household locations for cases, τ reflects the spatial scale of the avenues. We estimated τ with a moving window of 50 m computed every 10 m at distances starting at 420 m (because 5% of inter-avenue centroids fell below this value) to 2,500 m (the approximate width of Uvira). We calculated 95% CIs by using the 2.5th and 97.5th quantiles from 1,000 bootstrap replicates. We evaluated τ over a 5-day window, which included the date of case presentation, and a 4-day window, which excluded the date of case presentation, to provide a more realistic response on day 5 (*5*). To smooth the artifactual fluctuations resulting from the resolution of data and the smaller sample size of annual datasets, we calculated a moving average over the previous 10 m. We defined the high-risk zone around incident cases as the radius up to which the moving average's lower 95% CIs crossed 1.0 for ≥30 consecutive meters. We defined the elevated-risk zone around incident cases as the radius up to which the moving average point estimate crossed 1.0 for ≥30 consecutive meters. To explore the potential bias from using centroids compared with household locations, we conducted a simulation study where we randomly assigned household locations within each case-patient's avenue and then estimated τ by using a lower distance range (75–2,500 m).

Methods for τ Statistic

$\hat{\tau}(d_1, d_2)$ as an RR is approximated by dividing the odds that cases within the band are transmission-related $\hat{\theta}(d_1, d_2)$ by the same odds among cases in the general population (*3*,*5*,6), regardless of distance $\hat{\theta}(0, \infty)$.

The τ equation is: $\hat{\tau}(d_1, d_2) = \frac{\hat{\theta}(d_1, d_2)}{\hat{\theta}(0, \infty)}$

The odds for numerator $\hat{\theta}(d_1, d_2)$ are calculated as: $\hat{\theta}(d_1, d_2) = \frac{\sum i \sum j * I_1(i,j)}{\sum i \sum j * I_2(i,j)}$

The numerator tallies the number of case pairs (i, j) within the given distance band that are transmission-related (within 0–4 days), using indicator variable $I_1(i, j)$ = 1 for notation. The

denominator tallies the number of case pairs (i, j) within the given distance band that are not transmission-related (occurring after 4 days), using indicator variable $I_2(i,j) = 1$ for notation. The equivalent odds $\hat{\theta}(0,\infty)$ is estimated for the entire population.

Simulations to Compare Centroid-Geotagged Cases and Cases with Simulated Individual Household Locations

The case data used in this study are geocoded by X, Y coordinates, indicating 216 avenues (or streets) within the centroid belonging to a residence (Appendix Figure 1). In this simulation, we assessed whether using centroids versus simulated individual household locations affected trends in the τ statistic and to what extent.

Simulation Methods

We used the dataset of 1,493 rapid diagnostic tests (RDTs) that showed positive cholera cases from 2016–2020, displaying those results in space and time (Appendix Figure 2). The X, Y coordinates in this dataset were perturbed randomly by adding a random normal distribution that had an arbitrarily defined SD of 100. The points were plotted as maps to visually compare the spatial spread of cases between datasets 1 and 2 (Appendix Figure 3). The main τ analysis was run for each dataset. This produced the RR and 95% CI (τ statistic) of the next RDT-positive case being within a specific distance to another case compared with the risk of the case occurring anywhere else during days 0–4. A moving average was applied in distance spans of 10 m, 25 m, and 50 m to smooth fluctuations. To assess the similarity between the datasets, the moving average trend lines were evaluated visually by graphing and by comparing Pearson correlations.

Findings and Interpretation

The 2 datasets showed similar τ trends (Appendix Figure 4). Both the lower CIs of the moving average τ and the moving average τ point estimates (where τ consecutively crossed 1.0 for ≥30 consecutive meters) differed between the centroid and household datasets (Appendix Table 1). The Pearson correlation coefficients for the moving average τ point estimates were significant and nearly identical.

Overall, the centroid dataset showed a similar descending trend in risk over distance, central tendencies, and correlation coefficients compared with the simulated household dataset. The centroid dataset however showed 8.3% lower τ threshold estimate for the moving average τ

point estimate and 21.9% lower 95% CI moving average than the household simulation dataset. The simulated households compared to the centroid dataset had a higher maximum moving average τ estimate (equivalent to 2.0<RR<2.5) from 75–275 m (a distance segment that was unmeasured in the centroid dataset).

Software

Analyses were performed in R software version 4.1.2 (The R Project for Statistical Computing, https://www.r-project.org) using the rsatscan version 1.0.5 (https://github.com/Kenkleinman/rsatscan) and the IDSpatialStats version 0.3.12 (https://github.com/HopkinsIDD/IDSpatialStats) (*6*) R packages. rsatscan is used in tandem with SaTScan software version 10.0.2 (https://www.satscan.org) to calculate the space-time scan statistics.

References

1. Cleary E, Boudou M, Garvey P, Aiseadha CO, McKeown P, O'Dwyer J, et al. Spatiotemporal dynamics of sporadic Shiga toxin-producing *Escherichia coli* enteritis, Ireland, 2013–2017. Emerg Infect Dis. 2021;27:2421–33. PubMed https://doi.org/10.3201/eid2709.204021

2. Kulldorff M, Heffernan R, Hartman J, Assunção R, Mostashari F. A space-time permutation scan statistic for disease outbreak detection. PLoS Med. 2005;2:e59. PubMed https://doi.org/10.1371/journal.pmed.0020059

3. Lessler J, Salje H, Grabowski MK, Cummings DAT. Measuring spatial dependence for infectious disease epidemiology. PLoS One. 2016;11:e0155249. PubMed https://doi.org/10.1371/journal.pone.0155249

4. Azman AS, Rudolph KE, Cummings DAT, Lessler J. The incubation period of cholera: a systematic review. J Infect. 2013;66:432–8. PubMed https://doi.org/10.1016/j.jinf.2012.11.013

5. Azman AS, Luquero FJ, Salje H, Mbaïbardoum NN, Adalbert N, Ali M, et al. Micro-hotspots of risk in urban cholera epidemics. J Infect Dis. 2018;218:1164–8. PubMed https://doi.org/10.1093/infdis/jiy283

6. Giles JR, Salje H, Lessler J. The IDSpatialStats R Package: quantifying spatial dependence of infectious disease spread. R J. 2019;11:308–27. https://doi.org/10.32614/RJ-2019-043

Appendix Table 1. Differences in points where τ crosses RR = 1.0 for ≥30 consecutive meters consecutively*

Dataset	Min τ	Max τ	Mean τ	Moving average τ<1.0 (>30m)	Moving average τ LCI <1.0 (>30m)	Pearson correlation coefficient (95% CI)
Centroid	0.52	3.01	1.01	1,665 m	1,105 m	−0.87 (−0.89 to −0.85)
Simulated household†	0.55	2.40	1.05	1,815 m	1,415 m	−0.88 (−0.90 to −0.86)

*LCI, lower confidence interval; Max, maximum; Min, minimum; τ, tau statistic.
†Household locations were simulated and compared with the centroid dataset.

Appendix Table 2. Sensitivity analysis: statistically-significant spatiotemporal clusters of suspected cholera cases detected through annual scanning at the avenue level, Uvira, Democratic Republic of the Congo, 2016—2020

Year	Cluster No.	Cases observed: expected	Population at-risk	RR	Cluster radius (meters)	Cluster start date (mm/dd)	Cluster duration (d)
2016	1	57:5	177,122	10.8*	378	04/07	15
	2	51:4	187,076	12.1*	647	03/24	11
	3	45:6	183,225	7.2*	1,557	08/06	17
	4	27:3	120,498	8.4*	368	04/09	13
	5	40:9	147,424	4.6*	709	07/22	30
	6	18:2	29,390	7.8*	436	02/18	40
2017	1	130:13	148,014	10.8*	908	08/07	43
	2	91:16	150,104	5.9*	897	08/19	52
	3	39:6	88,959	6.6*	704	08/29	32
	4	23:2	134,147	10.6*	378	12/24	7
	5	26:5	143,948	5.2*	1,001	08/23	16
	6	9:1	42,275	17.3*	331	02/14	5
2018	1	50:3	130,673	15.3*	963	10/26	12
	2	24:2	134,311	15.1*	397	01/01	5
	3	61:15	132,515	4.2*	906	07/29	56
	4	44:10	128,631	4.5*	708	08/21	38
	5	18:3	70,142	5.9†	653	10/30	21
	6	9:1	52,203	14.4†	477	02/17	5
2019	1	50:4	93,453	14.3*	831	09/10	18
	2	30:2	21,965	13.9*	0	09/01	48
	3	47:7	105,035	7.1*	524	04/27	31
	4	48:10	115,699	5.0*	836	09/07	41
	5	36:8	120,197	4.7*	995	06/08	31
	6	14:2	40,341	7.4†	626	06/23	22
	7	6:0	45,292	32.2†	350	09/20	1
2020	1	105:17	159,204	6.7*	860	07/29	59
	2	59:11	141,671	5.8*	488	05/31	41
	3	38:5	106,256	8.6*	1,121	02/20	23
	4	57:13	155,765	4.6*	395	05/30	46
	5	49:13	120,618	3.9*	490	07/27	59
	6	39:10	159,261	4.0*	959	05/30	34
	7	15:2	44,366	10.1*	468	09/10	18

*p-value <0.001
† p-value <0.05
The p-value indicates the statistical significance of clusters derived from Monte Carlo simulations.
RR, relative risk.

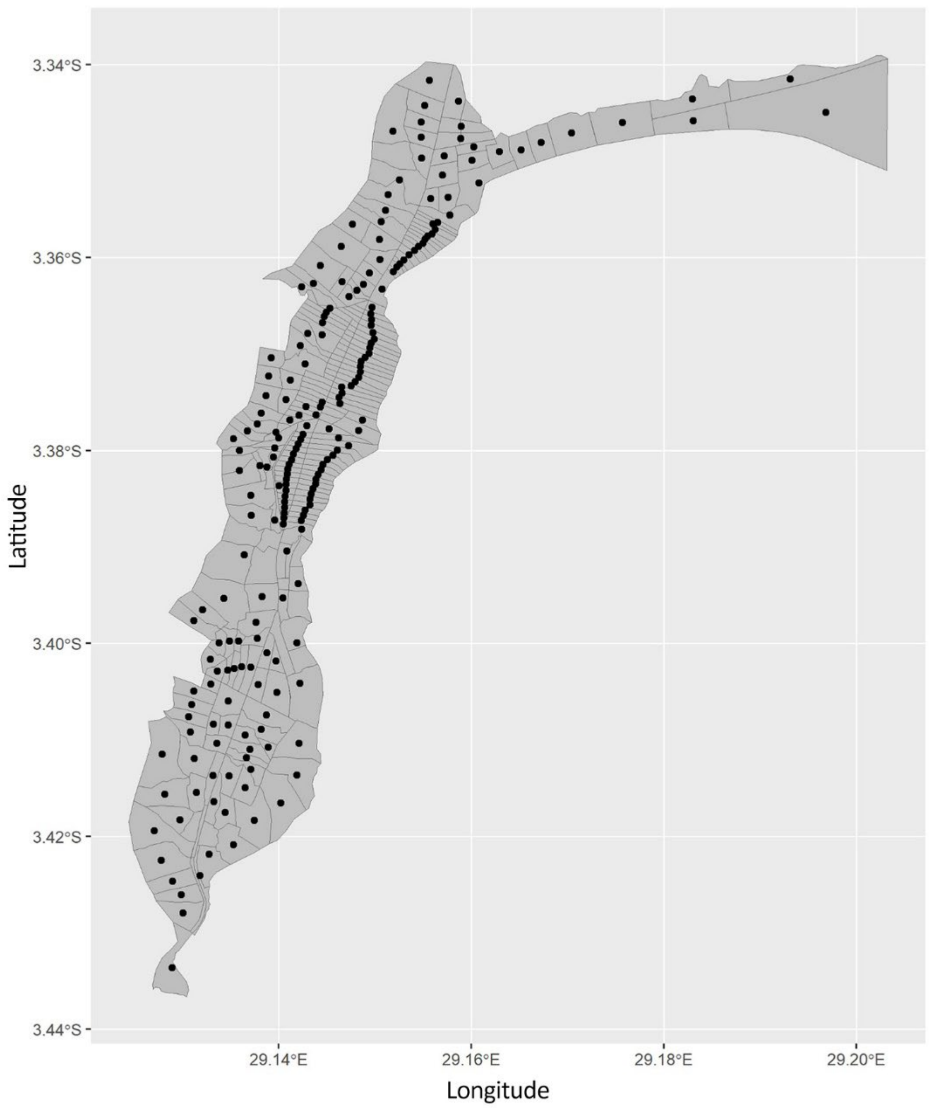

Appendix Figure 1. Information relevant to an analysis of spatiotemporal modeling of cholera, Uvira, Democratic Republic of the Congo, 2016–2020. Map of the centroid locations and borders of Uvira's 216 avenues.

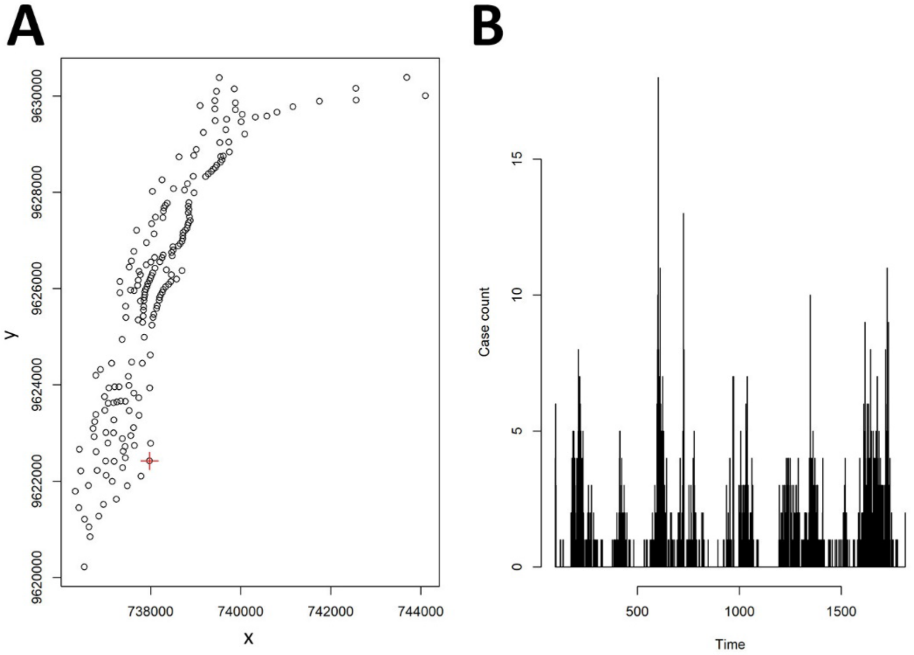

Appendix Figure 2. Information relevant to an analysis of spatiotemporal modeling of cholera, Uvira, Democratic Republic of the Congo, 2016–2020. Uvira 2016–2020 dataset of rapid diagnostic positive cases with avenue centroids of cases *(index case in red)*.

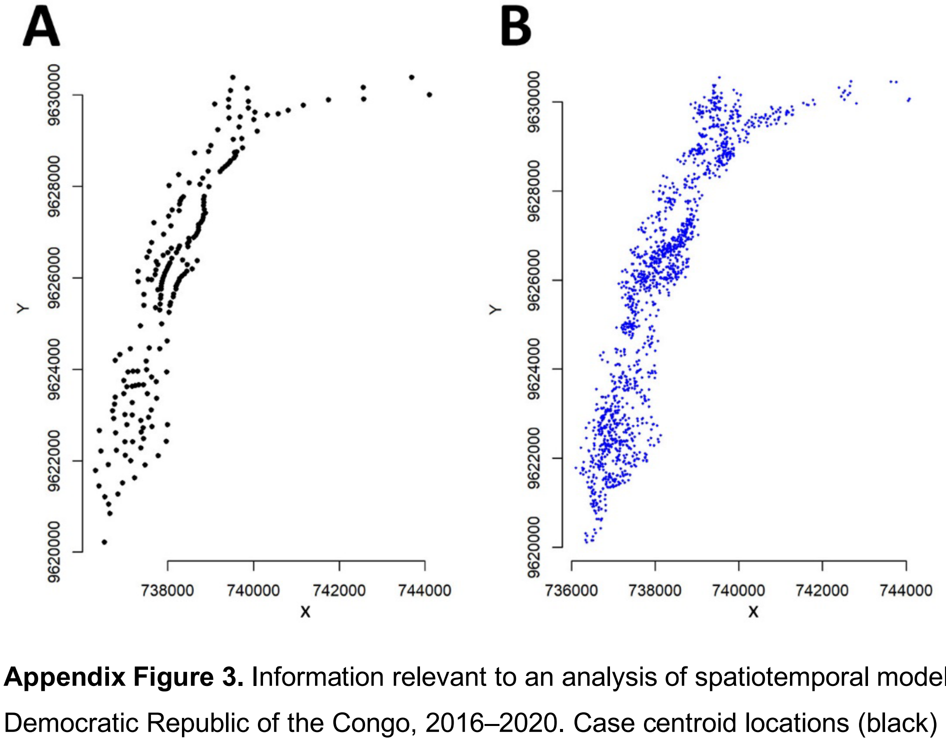

Appendix Figure 3. Information relevant to an analysis of spatiotemporal modeling of cholera, Uvira, Democratic Republic of the Congo, 2016–2020. Case centroid locations (black) and simulated household locations (blue).

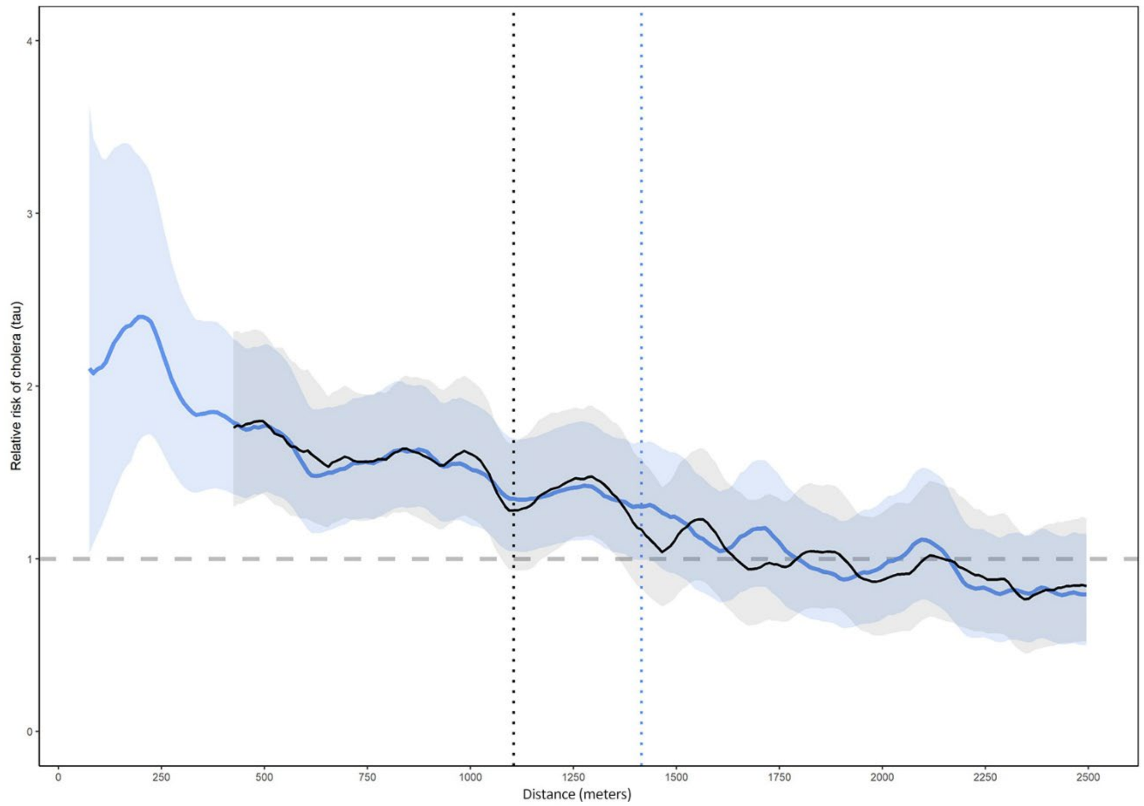

Appendix Figure 4. Information relevant to an analysis of spatiotemporal modeling of cholera, Uvira, Democratic Republic of the Congo, 2016–2020. Moving average of point estimates and 95% confidence intervals for tau τ statistic for RDT-positive cholera cases (75–2500 m) of the centroids (black, starting at 420 m) and the household locations (blue, starting at 75 m). The dashed line is where the lower confidence interval for the moving average crosses 1.0 for ≥30 consecutive meters consecutively.

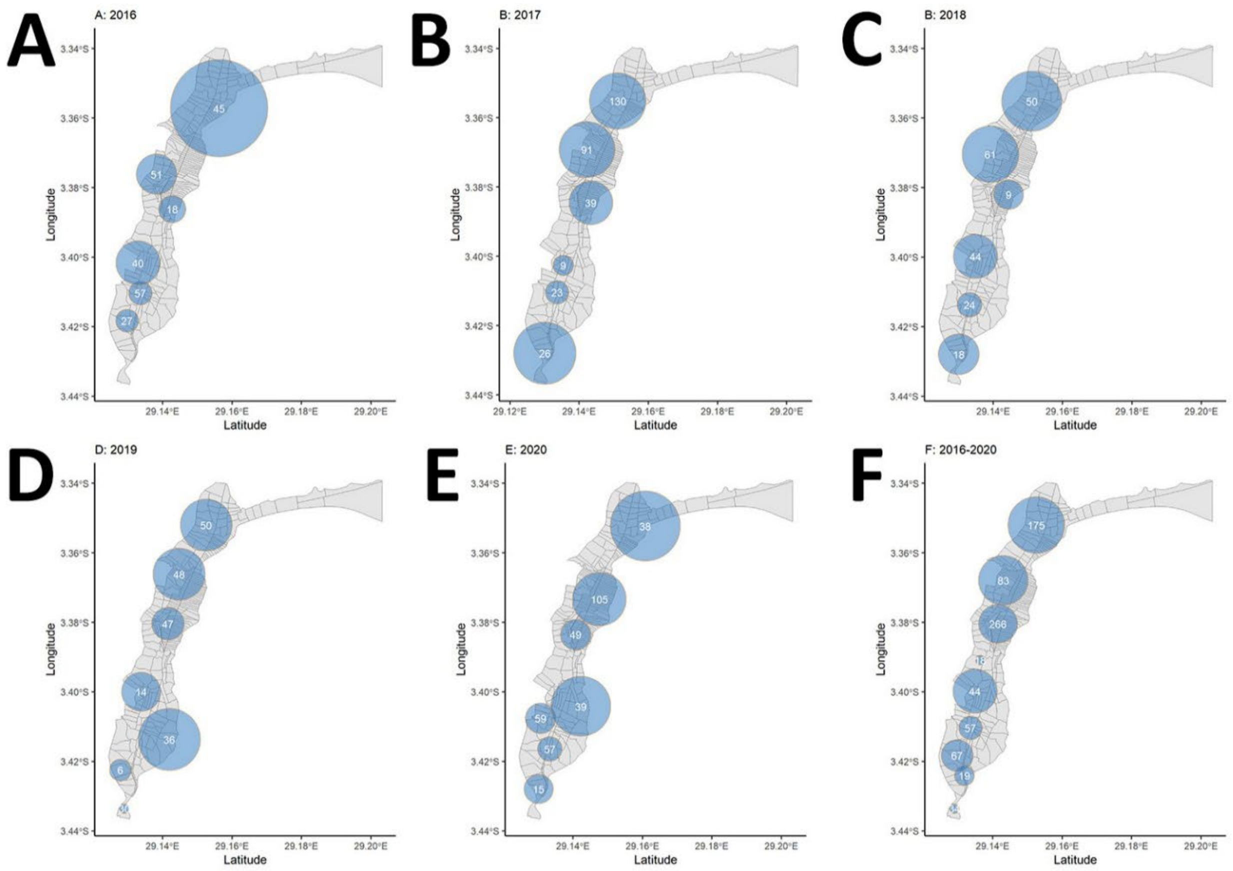

Appendix Figure 5. Information relevant to an analysis of spatiotemporal modeling of cholera, Uvira, Democratic Republic of the Congo, 2016–2020. Sensitivity analyses of prospectively detected spatiotemporal clusters of suspected cholera cases, 2016–2020. days. All scans had a maximum spatial window of 10% of the geographic area. The size of the orange circle depicts the radius with the number of suspected cases (in white). A–E depict scans with a temporal window of 7–60 days and F depicts a scan with a temporal window of 7–365. A) 2016; B) 2017; C) 2018; D) 2019; E) 2020; F) 2016–2020.

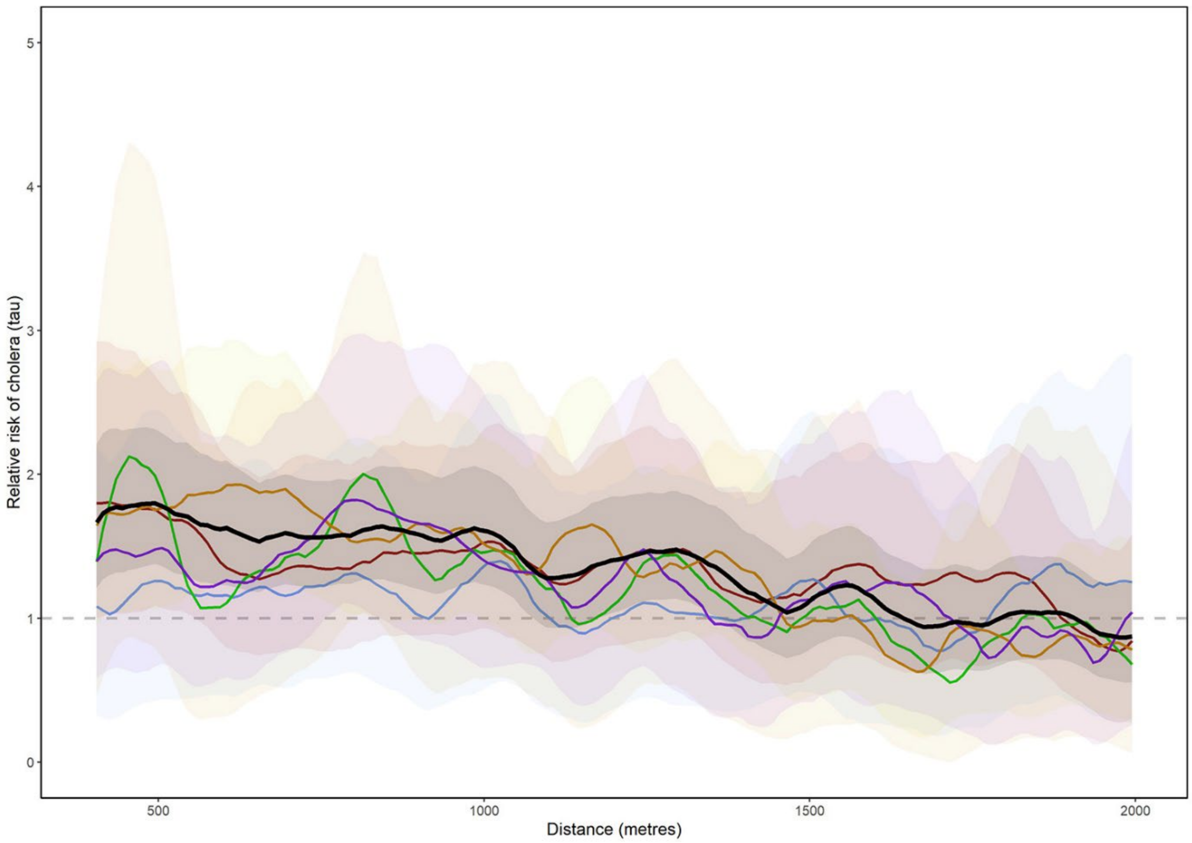

Appendix Figure 6. Information relevant to an analysis of spatiotemporal modeling of cholera, Uvira, Democratic Republic of the Congo, 2016–2020. Annual and aggregated moving average estimates of τ (relative risk) and 95% CIs (solid line and shading) for days 0–4. 2016–2020 in black, 2016 in purple, 2017 in orange, 2018 in green, 2019 in blue, 2020 in red.
